# Validity of the Polar H7 Heart Rate Sensor for Heart Rate Variability Analysis during Exercise in Different Age, Body Composition and Fitness Level Groups

**DOI:** 10.3390/s21030902

**Published:** 2021-01-29

**Authors:** Adrián Hernández-Vicente, David Hernando, Jorge Marín-Puyalto, Germán Vicente-Rodríguez, Nuria Garatachea, Esther Pueyo, Raquel Bailón

**Affiliations:** 1 Growth, Exercise, NUtrition and Development (GENUD) Research Group, University of Zaragoza, 50009 Zaragoza, Spain; jmarinp@unizar.es (J.M.-P.); gervicen@unizar.es (G.V.-R.); nugarata@unizar.es (N.G.); 2 Department of Physiatry and Nursing, Faculty of Health and Sport Science (FCSD), University of Zaragoza, 22002 Huesca, Spain; 3 Red Española de Investigación en Ejercicio Físico y Salud en Poblaciones Especiales (EXERNET), Spain; 4 BSICOS, Aragón Institute for Engineering Research (I3A), IIS Aragón, University of Zaragoza, 50015 Zaragoza, Spain; dhernand@unizar.es (D.H.); epueyo@unizar.es (E.P.); rbailon@unizar.es (R.B.); 5 CIBER de Bioingeniería, Biomateriales y Nanomedicina (CIBER-BBN), 50009 Zaragoza, Spain; 6 Centro de Investigación Biomédica en Red de Fisiopatología de la Obesidad y Nutrición (CIBER-Obn), 28029 Madrid, Spain; 7 Instituto Agroalimentario de Aragón-IA2- CITA-Universidad de Zaragoza), 50013 Zaragoza, Spain

**Keywords:** electrocardiography, wearable devices, HRV analysis, cluster analysis, exercise test

## Abstract

This work aims to validate the Polar H7 heart rate (HR) sensor for heart rate variability (HRV) analysis at rest and during various exercise intensities in a cohort of male volunteers with different age, body composition and fitness level. Cluster analysis was carried out to evaluate how these phenotypic characteristics influenced HR and HRV measurements. For this purpose, sixty-seven volunteers performed a test consisting of the following consecutive segments: sitting rest, three submaximal exercise intensities in cycle-ergometer and sitting recovery. The agreement between HRV indices derived from Polar H7 and a simultaneous electrocardiogram (ECG) was assessed using concordance correlation coefficient (CCC). The percentage of subjects not reaching excellent agreement (CCC > 0.90) was higher for high-frequency power (P_HF_) than for low-frequency power (P_LF_) of HRV and increased with exercise intensity. A cluster of unfit and not young volunteers with high trunk fat percentage showed the highest error in HRV indices. This study indicates that Polar H7 and ECG were interchangeable at rest. During exercise, HR and P_LF_ showed excellent agreement between devices. However, during the highest exercise intensity, CCC for P_HF_ was lower than 0.90 in as many as 60% of the volunteers. During recovery, HR but not HRV measurements were accurate. As a conclusion, phenotypic differences between subjects can represent one of the causes for disagreement between HR sensors and ECG devices, which should be considered specifically when using Polar H7 and, generally, in the validation of any HR sensor for HRV analysis.

## 1. Introduction

Heart rate (HR) variability (HRV) is the oscillation in the intervals between consecutive heartbeats (RR intervals) [1]. In the last decades, the use of HRV has been popularized, since it allows assessing cardiac autonomic modulation using simple and non-invasive techniques. In general, lower HRV has been associated with poorer prognosis in different clinical conditions, while higher HRV, especially regarding high-frequency oscillations, has been associated with better health. In particular, reduced HRV has been reported in several cardiovascular diseases and has been used for risk stratification, confirming its value as a predictor of total and cardiac mortality [2,3,4]. Also, low HRV has been described in a wide range of non-cardiovascular diseases, including psychiatric disorders such as depression, anxiety or schizophrenia [5].

Environmental and behavioral factors influence HRV [6]. In psychology, HRV is commonly used due to its modulation by mood states, emotions or cognitive capacity [5]. Likewise, HRV is a useful measurement in the sports field since it is sensitive to changes in fitness, fatigue and performance [7]. Healthy habits like exercise, balanced diet, mindfulness or psychological interventions have been shown to increase HRV measures indexing vagal function [5,6]. Additionally, non-modifiable factors, such as age or sex, also influence the autonomic regulation of the heart, with HRV having been reported to progressively decline with age and to present enhanced high-frequency oscillations and attenuated low-frequency oscillations in females as compared to males [8]. Because of these high inter- and intra-individual variations in HRV, it is key to use wearable devices validated for HRV analysis that can allow for precise interpretation of cardiac responses to different autonomic states.

A recent systematic review and meta-analysis showed that HRV measurements derived from portable devices are generally accurate when compared to lab-based electrocardiogram (ECG) [9]. Given the low cost of HR monitors it is not surprising that they are widely used by practitioners and researchers. Particularly, Polar Electro Oy (Kempele, Finland) is one of the most well-established brands in HR monitoring, with Polar H7/H10 HR sensors having been validated both at rest and during exercise [10,11,12]. Nevertheless, previous Polar validation studies have been carried out in small groups of young, lean, healthy and physically fit volunteers [10,11,12]. However, the device is commonly used by individuals with various phenotypic characteristics, regardless of how these may affect the accuracy of the measurements [13,14]. In the meta-analysis described in [9], the absolute error of portable devices was found to vary with the evaluated HRV metric, tilt/recovery position and the percentage of women in the study sample. The characteristics of the subjects and their influence on the measurements provided by portable devices have not been analyzed yet.

The ECG records the electrical activity of the heart using electrodes placed on the surface of the body. Therefore, differences between measurements from HR sensors and ECG could vary depending on the characteristics of the population under study. To start with, the age-related myocardial fibrosis present in the cardiac tissue, the amount of subcutaneous fat or the electrode placement are expected to affect the voltage tracings [15]. Additionally, voltage will be influenced by ventricular size or mass, as observed when comparing trained athletes with non-athletes [16]. Accordingly, some groups of subjects such as men, athletes or black/African have been reported to have higher QRS voltage and, consequently, RR intervals become easier to be detected [16]. On the other hand, obesity, older age and sedentary lifestyle may cause lower voltage, which may result in lower accuracy of portable devices. In particular, under such circumstances, some heart beats can be misdetected and, while this may not considerably affect mean HR, it may notably hamper HRV assessment. For these reasons and in light of previous studies, we hypothesized that when the quality of the Polar H7 ECG is compromised by high noise during intense exercise and/or by specific phenotypic characteristics of the subjects, HRV measures can be distorted, particularly those related to high-frequency power [17].

The purpose of the present study was to evaluate the validity of HRV analysis derived from RR intervals recorded by Polar H7 HR sensor at rest and during exercise and recovery in different phenotype groups based on age, body composition and fitness level.

## 2. Materials and Methods

### 2.1. Subjects

A total of sixty-seven males agreed to participate in the study. The sample consisted of three groups of volunteers: 22 young adults (20–30 years old), 22 middle-aged adults (40–50 years old) and 23 older adults (60–70 years old). Only subjects within the predefined age ranges were included in the study. Subjects were excluded from the study if they were going through an acute disease, were suffering from heart diseases (e.g., heart failure or atrial fibrillation), were on cardiac medication or presented any clinical condition contraindicating physical exercise. However, subjects who were overweight, sedentary or suffering from chronic diseases such as hypertension, diabetes or hypercholesterolemia were included in the study, because of their high prevalence in the society. Table 1 shows the descriptive characteristics of the three age groups. The study was approved by the ethical committee for clinical research of Aragón (ID of the approval: PI17/0409), and was conducted by adhering to the Declaration of Helsinki. After a clear explanation of the potential risks of the study, all volunteers provided written informed consent.

### 2.2. Procedure

All subjects completed one test session. Prior to the test, they were asked to adhere to the following instructions [18]: (1) avoid exercise or strenuous physical activity the day before the test; (2) drink plenty of fluids over the 24-h period preceding the test; (3) get an adequate amount of sleep (6–8 h) the night before the test; (4) avoid substances such as tobacco, alcohol or stimulants (caffeine, theine, taurine, etc.) in the 8 h before the test; (5) avoid food intake for 3 h prior to performing the test; and (6) wear comfortable, loose-fitting clothing. Subjects’ skin was prepared by using a razor to remove any hair from the electrode sites, cleaning the skin with alcohol and drying it with a gauze. A 12-lead high-resolution Holter ECG was acquired, with the 10 electrodes placed as indicated by the manufacturer (H12+, Mortara Instrument, Milwaukee, WI, USA), ensuring that they did not interfere with the HR sensor strap (Polar H7, Polar Electro Oy).

**Figure 1:**
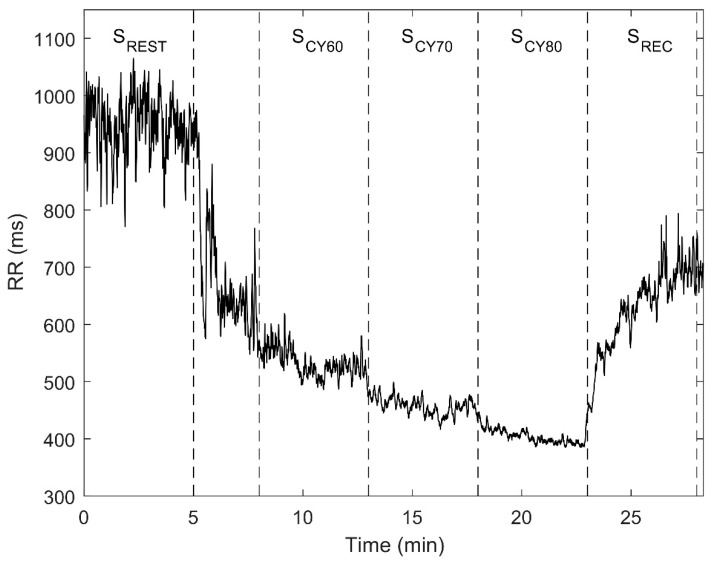
Example of the RR intervals for one subject throughout the entire test. Dotted lines separate the different test segments: resting (S_REST_), cycling (S_CY_) and recovery (S_REC_). S_CY_ was divided into three stages corresponding to 60, 70 and 80% of HRmax, denoted as S_CY60_, S_CY70_ and S_CY80_, respectively.

The test was conducted in an environmentally controlled room (22–23 °C), between 16:00–20:00, and was divided into 3 consecutive segments: resting (S_REST_), cycling (S_CY_) and recovery (S_REC_). During S_REST_, volunteers were monitored while seated at rest for 5 min, without any movement or talking. A period of 2–3 min was established to change from the chair to the cycle-ergometer, namely from S_REST_ to S_CY_, during which the subject rode the electrically braked cycle-ergometer (Ergoselect 200 K, Ergoline; Bitz, Germany) at 50 W workload and chose a cadence which was maintained during the entire test according to the workload and cadence displayed in the cycle-ergometer screen. S_CY_ was a submaximal cycle-ergometer test divided into three stages lasting 5 min each. In order to avoid a maximal exercise test, the maximum heart rate (HRmax) was estimated for each subject by using the formula defined by Tanaka et al. HRmax = 208 − 0.7 ∗ age (years) [19]. Workload was adjusted during each stage to 60, 70 and 80% of HRmax, with these stages denoted as S_CY60_, S_CY70_ and S_CY80_, respectively. Finally, during S_REC_, volunteers remained seated again for 5 min without any movement or talking. Figure 1 shows an example of the temporal evolution of RR intervals from a subject throughout the entire test.

### 2.3. Data Recording

Subjects self-reported their birth date, current diseases and medication. The anthropometric characteristics of the subjects were assessed. Stature was measured to the nearest 0.001 m using a portable stadiometer (SECA 225, Hamburg, Germany), with subjects standing with their scapula, buttocks and heels resting against a wall, the feet with the heels touching, forming a 45° angle and the head in the Frankfort’s plane. A portable body composition analyzer (TANITA BC-418MA; Tanita Corp., Tokyo, Japan) was used to measure the body mass to the nearest 0.1 kg, with underwear and after urination. TANITA BC-418MA was also used to estimate the percentage of body fat and trunk fat (r = 0.87, *p* < 0.001 vs. dual-energy X-ray absorptiometry) [20]. Body mass index (BMI) was calculated dividing weight in kilograms by height in squared meters.

Beat-to-beat RR intervals with 1-ms resolution were obtained using a Polar V800 HR monitor simultaneously with a Polar H7 chest Soft Strap (Polar Electro Oy, henceforth referred to as PolarH7). Concomitantly, a 12-lead ECG was recorded at a sampling rate of 1000 Hz using a high-resolution Holter device (H12+, Mortara Instrument, henceforth referred to as ECG and used here as a reference).

VO_2_max can be estimated from submaximal exercise tests, a safe and feasible method showing good validity against maximal tests (correlation coefficients: 0.69 to 0.98) [21]. Rather than commonly used tests with stages of short or variable duration, an ad-hoc test with 5-min stages was defined to allow reliable estimation of the low-frequency power of HRV. This enabled assessment of HRV response to increased sympathetic activity with each cycling stage [22]. Cardiorespiratory fitness was assessed using the approach of “Physical Work Capacity” (PWC). PWC in watts was measured during S_CY80_ of the submaximal cycle-ergometer test and was subsequently divided by the subject’s body weight (PWC_80%_ in W/kg). Alternatively to the use of fixed HR thresholds, this method incorporates the age-dependent decline of HRmax [23,24] and has been previously used as an objective assessment of cardiorespiratory fitness [25,26].

### 2.4. Data Analysis and Processing

Raw RR interval time series, RR_P_ (i), recorded by PolarH7 were downloaded from the “Polar Flow” web platform. RR interval time series from the ECG, RR_E_ (i), were extracted using a multi-lead approach by a wavelet-based detector [27] with optimized parameters for noisy environments as described in [28]. Each beat detection was manually verified by an operator with a dedicated interface.

The delay between the RR interval series RR_P_ (i) and RR_E_ (i) was estimated as the time lag maximizing their cross-correlation over the first 3 min of the test when the subject is relaxed. Then, both series were synchronized by compensating for this delay. Since the two RR interval series can have different lengths, due to, e.g., wrong or missed beat detections in the Polar data, an algorithm was developed to match the RR intervals from both series, thus allowing characterization of the agreement between the paired series RR_P_ (ip) and RR_E_ (ip), where ip refers to the indices of beats that are matched in the two series.

### 2.5. Heart Rate Variability

HRV indices were obtained by algorithms specifically developed and previously published by our research group using MATLAB version R2017a (MATLAB, MathWorks Inc., Natick, MA, USA) [17,27,28,29,30].

#### 2.5.1. Temporal Domain

The following temporal HRV indices were studied [1]: mean HR (MHR), standard deviation of normal-to-normal RR intervals (SDNN) and root mean square of successive differences of adjacent normal-to-normal RR intervals (RMSSD). MHR was obtained as the inverse of the mean of the RR intervals. SDNN is considered a measure of the total power of HRV and was calculated from the standard deviation of the NN intervals, i.e., normal RR intervals after correcting for ectopic beats [29]. RMSSD is a measure of short-term variability and was computed by the root mean square of successive differences between adjacent NN intervals. These indices were obtained from RR_P_ (i) and RR_E_ (i) in each segment of the test.

#### 2.5.2. Frequency Domain

The instantaneous HR signal, $d_{HR}\left(n\right)$, was derived from both RR_P_ (i) and RR_E_ (i) and sampled at 4 Hz. The integral pulse frequency modulation (IPFM) model was used while dealing with the presence of ectopic beats [29]. This signal was high-pass-filtered (0.03 Hz) to remove the very low-frequency components, $d_{MHR}\left(n\right)$, and it was also corrected by it: $m\left(n\right)=\;(d_{HR}\left(n\right)-\;d_{MHR}\left(n\right))/d_{MHR}\left(n\right)$ [30].

**Figure 2:**
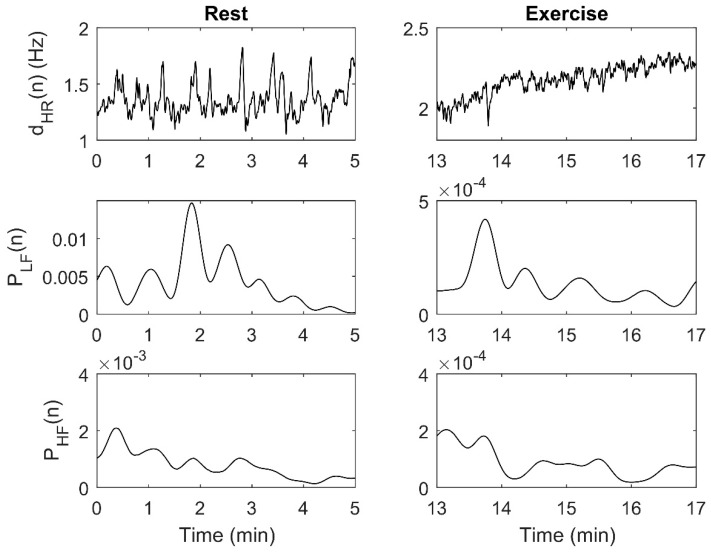
Example of $d_{HR}\left(n\right)$, P_LF_ (n) and P_HF_ (n) obtained from RR_E_ for one subject: Resting segment (**left**) and cycling segment (**right**). Note that the axes have different scales. $d_{HR}\left(n\right)$ = instantaneous HR signal; P_LF_ (n) = Instantaneous low-frequency power; P_HF_ (n) = Instantaneous high-frequency power; RR_E_ = RR intervals series from the ECG.

The smoothed pseudo Wigner–Ville distribution (SPWVD) was applied to m(n) to estimate its time-varying spectrum. Time and frequency smoothing windows were chosen as described in [17]. The instantaneous power in the low-frequency band, P_LF_ (n), was extracted integrating the SPWVD from 0.04 to 0.15 Hz for each time instant. The instantaneous power in the high-frequency band, P_HF_ (n), was computed in a band centered on the respiratory frequency with a bandwidth of 0.25 Hz. Figure 2 shows an example of $d_{HR}\left(n\right)$, P_LF_ (n) and P_HF_ (n) obtained from RR_E_. In some analyses, mean P_LF_ and P_HF_ were calculated from P_LF_ (n) and P_HF_ (n) for each segment of the test.

### 2.6. Statistical Analysis

The normality of data was checked with the Kolmogorov-Smirnov test. Since the data distribution violated the assumption of normality of the parametric tests, and such a condition was not achieved by commonly employed transformations, a non-parametric analysis was performed. Descriptive values are presented as mean ± standard deviation (SD) and HRV values are reported as median and interquartile range. Statistical analyses were performed using IBM SPSS (version 25; Chicago, IL, USA). The significance level was set at *p* ≤ 0.05.

Wilcoxon test for paired samples, the non-parametric equivalent of the paired samples *t*-test, was used to determine differences between the temporal domain HRV data obtained from PolarH7 and from ECG. The magnitude of the differences was calculated by determining the effect size (ES): $ES=Z/\sqrt{n}$ where *Z* represents the Z-score for the Wilcoxon statistic and *n* is the total number of observations [31]. Differences were considered small when ES < 0.2, small to medium when ES = 0.2–0.5, medium to large when ES= 0.5–0.8 and large when ES > 0.8 [32].

Lin’s concordance correlation coefficient (CCC) was used to study the agreement between the following PolarH7-derived and ECG-derived signals: RR (ip), P_HF_ (n) and P_LF_ (n). CCC determines how much the observed data deviate from the perfect concordance line at 45° on a square axis scatter plot [33]. CCC was evaluated in each segment (S_REST_, S_CY60_, S_CY70_, S_CY80_ and S_REC_). A CCC value greater than 0.90 was considered “excellent” [34] and the percentage of subjects with CCC values below this threshold was reported for each segment.

Cluster analysis was performed to identify groups of subjects with similar characteristics in terms of the following three variables of interest: age, body composition (trunk fat percentage) and fitness level (PWC_80%_). Trunk fat percentage was selected among all body composition variables, since it is the most specific to the electrode placement area. Following the methodology described in previous studies [35,36], two types of cluster analyses were combined: hierarchical clustering (Ward’s method) and k-means clustering. First, individual and multivariate outliers (according to Mahalanobis distance) were detected to reduce the sensitivity of the Ward’s method to outliers. Second, hierarchical cluster analysis was used, as the number of clusters in the data were unknown beforehand. Examination of dendrograms showed that a four-cluster solution produced good differentiation between groups. Finally, k-means cluster was performed with four possible solutions. Compared to hierarchical methods, k-means cluster analysis is considered less sensitive to outliers and has been found to result in greater within-cluster homogeneity and between-cluster heterogeneity [35].

To assess differences in the percentage of error for each HRV index between the four cluster groups, a Kruskal-Wallis test (non-parametric equivalent of one-way analysis of variance, ANOVA) with Bonferroni correction was performed. The Dunn-Bonferroni post hoc method was used for pairwise comparisons. The relative error in HRV indices was calculated as the absolute error of the PolarH7 with respect to the ECG measurement divided by the reference ECG measurement, e.g., $\left(SDNN_{ECG}-SDNN_{PolarH7}\right)/SDNN_{ECG}$, which was then multiplied by 100 to obtain the percentage of error (%Error). In the case of the frequency HRV variables, %Error was calculated from the mean value for each segment of the test. To evaluate the magnitude of the differences, ES was calculated as: $ES=H/(\left(n^{2}-1\right)/\left(n+1\right))$, where *H* stands for the Kruskal-Wallis test statistic and *n* is the total number of observations [31].

## 3. Results

Table 2 shows the descriptive characteristics of the 4 cluster groups, which were described as CLUSTER A (High PWC_80%_), CLUSTER B (Low PWC_80%_ and low age), CLUSTER C (Low PWC_80%_, high age and medium trunk fat percentage) and CLUSTER D (Low PWC_80%_, high age and high trunk fat percentage).

Table 3 shows the values of HRV indices obtained from PolarH7 and ECG. Mean P_LF_ and P_HF_ were calculated from P_LF_ (n) and P_HF_ (n) for each segment (differently from Table 4, where the instantaneous series were used). Wilcoxon test for paired samples revealed that P_HF_ and temporal domain HRV indices (MHR, SDNN and RMSSD) were lower at all cycling stages (S_CY60_, S_CY70_ and S_CY80_) when measured by PolarH7, with P_LF_ being lower at the highest intensity (S_CY80_) when measured by PolarH7. The magnitude of all these differences was small to medium, i.e., 0.2–0.5 according to the effect sizes.

Table 4 shows CCC values for RR (ip), P_LF_ (n) and P_HF_ (n) and outlines the percentage of subjects not reaching excellent agreement (CCC > 0.90) for each segment of the test. The number of subjects not reaching excellent agreement was clearly higher for P_HF_ (n) than for P_LF_ (n) (*χ* ^2^(degrees of freedom); *χ*^2^(1) = 45.52; *p* < 0.001), it increased with exercise intensity (*χ*^2^(2) = 38.47; *p* < 0.001) and was lower during exercise than during S_REC_ (*χ* ^2^(1) = 42.31; *p* < 0.001). When performing the analysis separately for each identified cluster, CLUSTER A obtained the highest CCC values, with CLUSTER D being the group with less subjects showing optimal agreement between devices in P_HF_ (n). Due to the presence of noise in the RR_P_ (i) series during S_REC_, the instantaneous power could not be properly extracted in 4 volunteers and the final sample for S_REC_ was N = 63.

Table 5 shows %Error for each HRV index. Kruskal-Wallis test demonstrated significant differences between clusters in P_HF_ at S_REST_ and during exercise (S_CY70_ and S_CY80_). With regards to temporal domain HRV indices, SDNN showed significant differences between groups at S_REST_ and during exercise (S_CY60_ and S_CY80_) and RMSSD showed significant differences at the highest intensities (S_CY70_ and S_CY80_). Both for P_HF_ and for temporal domain HRV indices, CLUSTER D was the group with the highest %Error. The magnitude of all these differences was small, i.e., <0.2 according to the effect sizes.

## 4. Discussion

In this study, HRV analysis from RR intervals provided by PolarH7 at rest and during various exercise intensities has been validated against the same analysis from a simultaneous ECG recording. Wilcoxon test showed a large number of significant differences between devices in HRV indices during exercise. However, the effect size was small to medium and of little practical relevance in the case of MHR. When observing RR (ip), P_LF_ (n) and P_HF_ (n) signals, the percentage of subjects not reaching excellent agreement between devices (CCC > 0.90) increased with exercise intensity and was higher for P_HF_ (n) than for P_LF_ (n). Cluster analysis revealed that phenotypic characteristics like age, body composition and fitness level influenced HRV measurements as well as the differences between PolarH7 and ECG. In particular, CLUSTER D, composed of subjects with low fitness level, high age and high trunk fat percentage, was the group with the lowest number of subjects obtaining excellent agreement between devices for P_HF_ (n) and with the highest %Error for time- and frequency-domain HRV indices.

The large number of significant differences in the temporal domain HRV indices (MHR, SDNN and RMSSD) between PolarH7 and ECG at all cycling stages could be due to a small but consistent difference between devices. Specifically, PolarH7 values were usually slightly lower than those measured by the ECG, which is supported by the obtained small to medium effect sizes. In the case of MHR, the values measured by the two devices for individual subjects were the same up to the 2nd-3rd decimal figure. Even if significant, such differences between devices may not be meaningful in practice, as differences of less than one beat per minute are below what has been reported as the smallest worthwhile change in previous studies [37].

Regarding analysis of the full paired series of RR intervals, excellent agreement between devices was found at rest, in accordance with previous validation studies of PolarH7/H10 HR sensors [10,12]. Also, our results confirmed that the agreement between devices decreased with the intensity of exercise, as previously reported [11,12]. Despite this reduction, Gilgen-Ammann et al. proposed Polar H10 as the gold standard for RR interval assessment during intense activities for HR and HRV evaluation [12]. It should be noted, however, that a reduced set of ten healthy, lean and physically fit volunteers was considered in [12], whereas here we investigated a larger set of volunteers with a broader range of ages, body compositions and fitness levels. This may explain why we found a more noticeable reduction in the agreement between devices during exercise.

Frequency-domain HRV indices, including P_LF_ and P_HF_, were not usually investigated in previous studies validating PolarH7/H10 HR sensors. Nevertheless, these frequency-domain signals were evaluated in the previous Polar RS800 model, reporting that differences between devices increased with exercise intensity and were higher for P_HF_ than for P_LF_ [17]. In the present study, P_LF_ showed excellent agreement at rest and during the whole exercise test, meaning that PolarH7 can follow HR oscillations up to 0.15 Hz. Still, the percentage of subjects reaching excellent agreement for P_LF_ at the highest intensities (81% with CCC > 0.9 at 80% of HRmax) was lower than in [17] (96% with Pearson correlation coefficient > 0.8 at 80–100% of VO_2_max), possibly due to the greater heterogeneity of the present population sample. In the case of P_HF_, we found that PolarH7 and ECG showed disagreement at the highest intensities, in accordance with results reported for Polar RS800, possibly due to a multifactorial etiology, including the higher respiratory frequency and higher noise level during exercise, the processing performed by Polar when a beat cannot be detected and the effect of the body characteristics of the subjects [17]. Since both P_HF_, reflecting vagal modulation of cardiac activity, and its highly correlated time-domain HRV measures, such as RMSSD [38], are commonly used to monitor the autonomic status before, during and after exercise [39], a note of caution on the interpretation of results obtained from PolarH7 is suggested, especially at exercise intensities greater than 70% of HRmax.

Our results from clustering analysis confirmed the hypothesis that the phenotypic characteristics of the subjects are one of the causes for the observed differences between PolarH7 and ECG devices [17]. As initially postulated, CLUSTER D, containing subjects with low fitness, high age and high trunk fat percentage, was the one showing the highest %Error for HRV indices, reaching 50% error in P_HF_ and 30% error in RMSSD for intensities greater than 70% of HRmax. Nevertheless, even in CLUSTER D, some subjects presented excellent agreement between devices, confirming that the characteristics of the subjects are not the only cause of disagreement. Future studies should clarify other possible reasons underlying the observed differences between devices.

**Figure 3:**
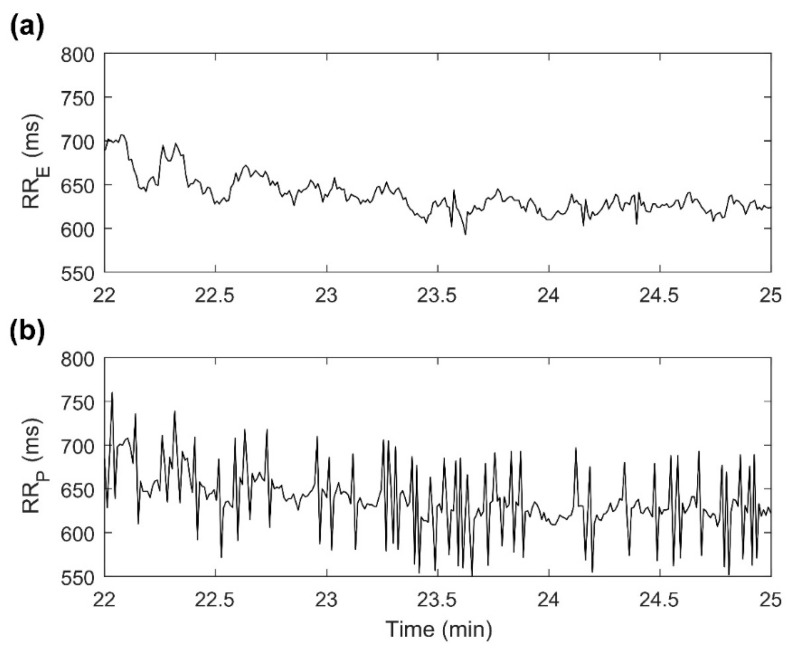
Example of RR intervals recorded by both devices during the recovery segment for one subject. (**a**) RR_E_ = RR intervals series recorded by ECG; (**b**) RR_P_ = RR intervals series recorded by PolarH7.

The recovery from exercise was the time period when the lowest CCC values were measured, especially for P_LF_ and P_HF_ signals. To our knowledge, this is the first time that PolarH7 validity has been analyzed during sitting recovery. The lack of agreement between devices could be due to the noisy signal recorded by PolarH7 in some volunteers, as illustrated in Figure 3. Consequently, despite the correction algorithms, 10% of P_LF_ signals and 27% of P_HF_ signals presented very low agreement (CCC < 0.1). Based on these results, assessment of HRV, particularly P_LF_ and P_HF_, during the recovery period may not be reliable if PolarH7 is used. This is in line with Schneider et al., who recommended evaluation of HR recovery rather than evaluating post-exercise HRV [7].

### Strengths and Limitations

The present study has several main strengths. One of them is the phenotypic variety of the 67 volunteers. HR sensor validation studies are often carried out in groups of 20, or even fewer, young, lean, healthy and physically fit volunteers [10,11,12], and in these conditions it may be easier to detect RR intervals. Therefore, our larger sample of 20- to 70-year-old subjects with varied physical conditions is much more heterogeneous and representative of HR sensor users. Secondly, our assessment of PolarH7 validity as a function of phenotypic characteristics, including age, is particularly relevant considering that the older population is growing all around the world and more so in Europe [40], with advanced age being associated with changes in body composition and reduced cardiorespiratory fitness [41,42]. Taking into account these associations, cluster analysis was used to evaluate how the concurrence of these characteristics in the volunteers could affect HRV measurements obtained from PolarH7. In third place, cardiorespiratory fitness and the excess of body fat are strong predictors of mortality and risk of cardiovascular disease, being age the main risk factor for multimorbidity [40,42]. Accordingly, it is of special interest to evaluate the validity of these devices in subjects with these phenotypic characteristics. As discussed in the introduction of the study, the applications of HRV in the evaluation and management of a wide range of diseases are growing. The use of these inexpensive and simple to use devices could be a very useful tool for E-health in primary care. HRV measures that can be reliably assessed by HR sensors like PolarH7 need to be established so that interpretations can be safely made. Last but not least, all our body measurements and signal recordings were performed in the laboratory, under homogeneous conditions, enabling the control of confounding factors and the reproducibility of the study.

On the other hand, some limitations need to be acknowledged. According to the meta-analysis by Dobbs et al., the degree of absolute error between portable devices and ECG measurements was larger among studies involving a greater number of female subjects [9]. Here, only men were studied so that sex was not a confounding variable. Further research over other populations including not only women but also black/African would allow confirming the results obtained by this study in wider populations. Another potential limitation is that PolarH7 has been superseded by the Polar H10 band. Even so, millions of users still wear a PolarH7 band and the performance of both bands seems to be similar during stationary exercise [43]. Future studies could extend the research here presented to the analysis of other HR sensors.

## 5. Conclusions

Three major findings have emerged from the present study. First, assessment of HR and HRV in a relatively large and heterogeneous sample has confirmed that PolarH7 can accurately measure mean HR and low-frequency oscillations (up to 0.15 Hz) of HR at rest and during exercise. However, disagreement between PolarH7 and ECG exists when evaluating high-frequency HR oscillations during moderate-to-high intensity exercise. Second, the validity of PolarH7 measurements during sitting recovery has been studied for the first time. The results of the present research support the notion that PolarH7 is appropriate to study HR recovery rather than post-exercise HRV. Third, clustering analysis shows that the agreement between PolarH7 and ECG devices varies depending on the characteristics of the subjects regarding age, body composition and fitness level. Our results point to the need of ensuring phenotypic variety in any validation studies of HR sensors.

## Figures and Tables

**Table 1:** Descriptive characteristics of the three age groups.

Outcome	Young Adults (*n* = 22)	Middle-Aged Adults (*n* = 22)	Older Adults (*n* = 23)
Age (years)	25.46 ± 2.85	43.17 ± 3.32	63.97 ± 2.79
Height (m)	1.75 ± 0.06	1.77 ± 0.06	1.71 ± 0.05
Weight (kg)	72.01 ± 11.92	78.19 ± 10.30	76.31 ± 7.76
BMI (m/kg^2^)	23.43 ± 2.95	25.02 ± 2.83	26.21 ± 2.84
Body fat (%)	15.25 ± 5.59	19.69 ± 5.65	23.38 ± 5.17
Trunk fat (%)	16.31 ± 6.32	21.29 ± 6.38	25.69 ± 6.42
PWC_80%_ (W/kg)	2.00 ± 0.64	2.01 ± 0.58	1.73 ± 0.65

Values are expressed as mean ± standard deviation (SD). BMI = Body mass index; PWC_80%_ = Physical Work Capacity at 80% of maximum HR (208 − 0.7 ∗ age in years) in watts/kg bodyweight.

**Table 2:** Descriptive characteristics of the four cluster groups.

Outcome	CLUSTER A (*n* = 19)	CLUSTER B (*n* = 13)	CLUSTER C (*n* = 18)	CLUSTER D (*n* = 17)	Main Effect	
					*p*	Effect Size
Age (years)	38.99 ± 13.31 ^B,D^	24.35 ± 2.20 ^A,C,D^	52.61 ± 10.20 ^B^	57.47 ± 12.23 ^A,B^	<0.001 *	0.586
Height (m)	1.77 ± 0.05 ^D^	1.72 ± 0.06	1.74 ± 0.06	1.72 ± 0.07 ^A^	0.020	0.149
Weight (kg)	72.65 ± 9.13	69.18 ± 12.27 ^D^	76.32 ± 7.13	82.72 ± 8.78 ^B^	0.007	0.182
BMI (m/kg^2^)	23.00 ± 2.24 ^D^	23.23 ± 2.77 ^D^	25.17 ± 1.40	28.04 ± 2.79 ^A,B^	<0.001 *	0.430
Body fat (%)	14.09 ± 4.11 ^C,D^	15.45 ± 4.54 ^D^	20.40 ± 2.36 ^A,D^	27.67 ± 2.42 ^A,B,C^	<0.001 *	0.743
Trunk fat (%)	14.76 ± 5.11 ^C,D^	16.64 ± 5.03 ^D^	22.19 ± 2.86 ^A,D^	30.69 ± 2.12 ^A,B,C^	<0.001 *	0.725
PWC_80%_ (W/kg)	2.73 ± 0.39 ^B,C,D^	1.61 ± 0.37 ^A^	1.79 ± 0.28 ^A,D^	1.35 ± 0.22 ^A,C^	<0.001 *	0.704

Values are expressed as mean ± standard deviation (SD). BMI = Body mass index; PWC_80%_ = Physical Work Capacity at 80% of HRmax (208 − 0.7 ∗ age in years) in watts/kg bodyweight. Clusters were based on: age, body composition (trunk fat percentage) and fitness level (PWC_80%_). * = Significant differences between clusters (*p* ≤ 0.05, Kruskal-Wallis test). ^A^ = Different to CLUSTER A; ^B^ = Different to CLUSTER B; ^C^ = Different to CLUSTER C; ^D^ = Different to CLUSTER D.

**Table 3:** HRV indices obtained from PolarH7 and ECG data (*n* = 67).

		PolarH7	ECG	*p*	ES
S_REST_	P_LF_ (e^−4^)	9.78 (3.88 to 24.74)	9.76 (3.85 to 24.78)	0.074	0.155
	P_HF_ (e^−4^)	5.77 (2.30 to 10.91)	5.74 (2.22 to 10.84)	0.067	0.159
	MHR (bpm)	62.55 (53.25 to 71.95)	62.84 (53.36 to 72.00)	<0.001 *	0.378
	SDNN (ms)	60.27 (40.50 to 75.22)	60.28 (40.33 to 75.26)	0.570	0.049
	RMSSD (ms)	39.48 (22.48 to 60.04)	39.26 (22.39 to 60.66)	0.336	0.083
S_CY60_	P_LF_ (e^−4^)	1.17 (0.62 to 2.01)	1.17 (0.65 to 2.23)	0.112	0.137
	P_HF_ (e^−4^)	0.51 (0.26 to 1.29)	0.86 (0.44 to 2.07)	<0.001 *	0.419
	MHR (bpm)	106.00 (100.45 to 112.91)	106.66 (100.75 to 113.17)	<0.001 *	0.433
	SDNN (ms)	15.94 (12.06 to 20.77)	16.21 (12.85 to 20.32)	0.010 *	0.222
	RMSSD (ms)	6.48 (4.34 to 8.51)	8.38 (5.67 to 10.65)	<0.001 *	0.482
S_CY70_	P_LF_ (e^−4^)	0.45 (0.21 to 0.87)	0.46 (0.22 to 0.88)	0.851	0.016
	P_HF_ (e^−4^)	0.30 (0.15 to 0.55)	0.41 (0.24 to 0.75)	<0.001 *	0.377
	MHR (bpm)	124.24 (115.77 to 130.89)	124.32 (115.76 to 130.92)	<0.001 *	0.482
	SDNN (ms)	10.22 (8.19 to 12.93)	10.48 (8.40 to 13.10)	0.001 *	0.280
	RMSSD (ms)	3.74 (2.96 to 4.91)	4.37 (3.64 to 6.68)	<0.001 *	0.443
S_CY80_	P_LF_ (e^−4^)	0.13 (0.09 to 0.23)	0.18 (0.10 to 0.26)	<0.001 *	0.364
	P_HF_ (e^−4^)	0.23 (0.14 to 0.36)	0.37 (0.25 to 0.66)	<0.001 *	0.362
	MHR (bpm)	141.01 (130.62 to 148.81)	141.09 (130.69 to 149.05)	<0.001 *	0.451
	SDNN (ms)	8.11 (6.20 to 9.92)	8.10 (6.34 to 10.65)	<0.001 *	0.378
	RMSSD (ms)	2.90 (2.32 to 3.90)	3.75 (3.16 to 5.52)	<0.001 *	0.405
S_REC_	P_LF_ (e^−4^)	4.75 (1.83 to 10.25)	4.59 (1.88 to 9.97)	0.881	0.015
	P_HF_ (e^−4^)	2.06 (0.65 to 4.71)	2.12 (0.82 to 4.70)	0.308	0.102
	MHR (bpm)	99.76 (90.76 to 112.15)	98.39 (90.12 to 111.35)	0.002 *	0.268
	SDNN (ms)	33.11 (23.27 to 58.30)	33.67 (23.04 to 57.40)	0.094	0.147
	RMSSD (ms)	12.87 (7.65 to 23.78)	12.90 (7.99 to 23.32)	0.603	0.046

Values are expressed as median and interquartile range. Segments are based on the test phases: resting (S_REST_), cycling (S_CY_) and recovery (S_REC_). S_CY_ was divided in three stages at 60, 70 and 80% of HRmax, denoted as S_CY60_, S_CY70_ and S_CY80,_ respectively. P_LF_ = low-frequency power; P_HF_ = high-frequency power; MHR = mean HR; SDNN = SD of the NN intervals; RMSSD = root mean square of successive differences between NN intervals. ES = Effect size. * = Significant differences between devices (*p* ≤ 0.05, Wilcoxon test for paired samples).

**Table 4:** Agreement between devices in: RR (ip), PLF (n) and PHF (n). CCC mean and percentage of subjects not reaching excellent agreement for each segment.

		S_REST_	S_CY60_	S_CY70_	S_CY80_	S_REC_
Whole sample (*n* = 67)	RR (ip)	0.9929 (1%)	0.9560 (6%)	0.9467 (13%)	0.9319 (16%)	0.9612 (14%)
	P_LF_ (n)	0.9885 (1%)	0.9713 (4%)	0.9677 (9%)	0.9106 (19%)	0.8251 (30%)
	P_HF_ (n)	0.9813 (3%)	0.9494 (13%)	0.8858 (27%)	0.6661 (60%)	0.5262 (75%)
CLUSTER A (*n* = 19)	RR (ip)	0.9970 (0%)	0.9844 (0%)	0.9472 (21%)	0.9243 (21%)	0.9778 (6%)
	P_LF_ (n)	0.9999 (0%)	0.9990 (0%)	0.9911 (5%)	0.9169 (16%)	0.7440 (47%)
	P_HF_ (n)	0.9982 (0%)	0.9645 (16%)	0.9316 (11%)	0.7970 (37%)	0.4859 (88%)
CLUSTER B (*n* = 13)	RR (ip)	0.9828 (8%)	0.8996 (15%)	0.8690 (23%)	0.9258 (23%)	0.9473 (15%)
	P_LF_ (n)	0.9423 (8%)	0.8544 (23%)	0.9757 (8%)	0.8601 (15%)	0.8838 (15%)
	P_HF_ (n)	0.9284 (8%)	0.8710 (23%)	0.9112 (23%)	0.7455 (62%)	0.7402 (54%)
CLUSTER C (*n* = 18)	RR (ip)	0.9943 (0%)	0.9363 (11%)	0.9665 (11%)	0.9035 (22%)	0.9615 (12%)
	P_LF_ (n)	0.9990 (0%)	0.9998 (0%)	0.9792 (6%)	0.9164 (28%)	0.7791 (31%)
	P_HF_ (n)	0.9909 (6%)	0.9670 (6%)	0.8906 (28%)	0.5914 (67%)	0.3843 (88%)
CLUSTER D (*n* = 17)	RR (ip)	0.9945 (0%)	0.9884 (0%)	0.9844 (0%)	0.9751 (0%)	0.9541 (24%)
	P_LF_ (n)	0.9999 (0%)	0.9996 (0%)	0.9233 (18%)	0.9361 (18%)	0.9045 (24%)
	P_HF_ (n)	0.9929 (0%)	0.9739 (12%)	0.8100 (47%)	0.5381 (76%)	0.5366 (65%)

Values are expressed as CCC mean and (percentage of subjects under 0.9 threshold). Segments are based on the test phases: resting (S_REST_), cycling (S_CY_) and recovery (S_REC_). S_CY_ was divided in three stages at 60, 70 and 80% of HRmax, denoted as S_CY60_, S_CY70_ and S_CY80_ respectively. The characteristics of each cluster were the following: CLUSTER A = high fitness; CLUSTER B = low fitness and low age; CLUSTER C = low fitness, high age and medium trunk fat percentage; CLUSTER D = low fitness, high age and high trunk fat percentage. RR (ip) = paired RR interval series; P_LF_ (n) = instantaneous low-frequency power; P_HF_ (n) = instantaneous high-frequency power.

**Table 5:** Percentage of error (%) for each HRV index and comparison between clusters.

		CLUSTER A (*n* = 19)	CLUSTER B (*n* = 13)	CLUSTER C (*n* = 18)	CLUSTER D (*n* = 17)	Main Effect	
						*p*	Effect Size
S_REST_	P_LF_	0.0 (−0.2 to 0.3)	0.2 (−0.3 to 0.6)	0.1 (−0.2 to 0.2)	0.3 (−0.1 to 0.4)	0.534	0.034
	P_HF_	−0.5 (−2.3 to 0.2)	−0.2 (−0.7 to 0.4)	−0.5 (−1.4 to 0.4)	0.5 (−0.3 to 2.0)	0.049 *	0.121
	SDNN	0.0 (−0.2 to 0.5) ^B^	−0.1 (−0.8 to 0.0) ^A,D^	0.0 (−0.2 to 0.2)	0.1 (0.0 to 0.2) ^B^	0.021 *	0.147
	RMSSD	−0.2 (−1.0 to 0.3)	−0.1 (−2.3 to 0.4)	−0.1 (−1.3 to 0.4)	0.2 (−0.2 to 2.3)	0.150	0.081
S_CY60_	P_LF_	0.8 (−0.7 to 18.7)	0.0 (−0.8 to 1.3)	0.4 (−4.0 to 3.0)	0.8 (−0.5 to 4.4)	0.444	0.041
	P_HF_	25.7 (10.3 to 48.3)	2.1 (−12.5 to 17.7)	21.7 (−2.4 to 44.6)	27.0 (10.9 to 65.6)	0.164	0.077
	SDNN	0.1 (−2.5 to 0.9) ^D^	−0.2 (−1.2 to 1.4)	1.2 (−0.1 to 4.2)	2.2 (1.1 to 8.5) ^A^	0.018 *	0.153
	RMSSD	14.4 (4.6 to 32.4)	1.8 (−3.2 to 14.6)	21.7 (4.2 to 29.8)	15.8 (7.9 to 44.1)	0.266	0.060
S_CY70_	P_LF_	1.6 (−0.5 to 7.5)	−0.2 (−2.8 to 0.9)	−0.4 (−1.7 to 2.1)	−0.6 (−7.8 to 2.8)	0.125	0.087
	P_HF_	9.2 (−6.2 to 50.8)	−0.9 (−42.6 to 25.6) ^C,D^	25.3 (17.5 to 57.2) ^B^	51.8 (14.5 to 71.7) ^B^	0.007 *	0.186
	SDNN	0.6 (−0.5 to 2.8)	0.2 (−1.4 to 1.6)	1.5 (−1.2 to 7.0)	2.3 (0.3 to 8.0)	0.104	0.093
	RMSSD	15.4 (−2.0 to 29.5)	0.6 (−27.9 to 19.3) ^D^	15.6 (14.2 to 36.5)	27.1 (14.2 to 56.0) ^B^	0.010 *	0.172
S_CY80_	P_LF_	1.5 (−2.6 to 32.0)	2.8 (−1.0 to 3.6)	2.7 (−1.1 to 12.6)	5.9 (0.3 to 16.1)	0.596	0.029
	P_HF_	31.6 (−27.4 to 87.7)	−9.0 (−113.1 to 56.4) ^D^	28.0 (8.5 to 45.2)	44.7 (37.2 to 77.7) ^B^	0.047 *	0.121
	SDNN	4.0 (−0.8 to 13.7)	−0.4 (−2.9 to 6.1)	1.1 (−0.3 to 4.1)	5.7 (1.6 to 14.3)	0.050 *	0.119
	RMSSD	19.2 (−9.9 to 67.0)	8.4 (−43.0 to 26.1) ^D^	19.0 (4.7 to 32.7)	32.9 (22.1 to 58.2) ^B^	0.028 *	0.137
S_REC_	P_LF_	−0.4 (−33.8 to 0.1)	0.5 (−0.5 to 6.2)	0.2 (−17.6 to 3.9)	0.2 (−4.9 to 12.7)	0.161	0.105
	P_HF_	−1.6 (−8.2 to 5.8)	1.6 (0.1 to 5.9)	−1.2 (−8.3 to 10.1)	9.3 (−0.2 to 37.2)	0.053	0.157
	SDNN	−0.1 (−0.9 to 0.1)	0.1 (−1.5 to 0.3)	−0.1 (−1.0 to 0.4)	0.0 (−1.8 to 0.7)	0.933	0.007
	RMSSD	−0.9 (−2.3 to 2.0)	0.1 (−9.8 to 3.0)	1.5 (−1.4 to 5.5)	2.2 (−2.9 to 11.5)	0.226	0.068

Percentage error (%) values are expressed as median and interquartile range. The characteristics of each cluster were the following: CLUSTER A = high fitness; CLUSTER B = low fitness and low age; CLUSTER C = low fitness, high age and medium trunk fat percentage; CLUSTER D = low fitness, high age and high trunk fat percentage. Segments are based on the test phases: resting (S_REST_), cycling (S_CY_) and recovery (S_REC_). S_CY_ was divided in three stages at 60, 70 and 80% of HRmax, denoted as S_CY60_, S_CY70_ and S_CY80_ respectively. P_LF_ = low-frequency power; P_HF_ = high-frequency power; SDNN= SD of the RR intervals; RMSSD = root mean square of successive differences between NN intervals. * = Significant differences between clusters (*p* ≤ 0.05, Kruskal-Wallis test). ^A^ = Different to CLUSTER A; ^B^ = Different to CLUSTER B; ^C^ = Different to CLUSTER C; ^D^ = Different to CLUSTER D.

## Data Availability

The datasets analyzed during the current study are available from the corresponding author on reasonable request.

## References

[1] Malik M., Camm A.J., Bigger J.T., Breithardt G., Cerutti S., Cohen R.J., Coumel P., Fallen E.L., Kennedy H.L., Kleiger R.E. (1996). Heart rate variability. Standards of measurement, physiological interpretation, and clinical use. Eur. Heart J..

[2] Spallone V., Ziegler D., Freeman R., Bernardi L., Frontoni S., Pop-Busui R., Stevens M., Kempler P., Hilsted J., Tesfaye S. (2011). Cardiovascular autonomic neuropathy in diabetes: Clinical impact, assessment, diagnosis, and management. Diabetes Metab. Res. Rev..

[3] Kleiger R.E., Miller J.P., Bigger J.T., Moss A.J. (1987). Decreased Heart Rate Variability and Its Association with Increased Mortality After Acute Myocardial Infarction. Am. J. Cardiol..

[4] Bilchick K.C., Fetics B., Djoukeng R., Gross Fisher S., Fletcher R.D., Singh S.N., Nevo E., Berger R.D. (2002). Prognostic value of heart rate variability in chronic congestive heart failure (Veterans Affairs’ Survival Trial of Antiarrhythmic Therapy in Congestive Heart Failure). Am. J. Cardiol..

[5] Kemp A.H., Koenig J., Thayer J.F. (2017). From psychological moments to mortality: A multidisciplinary synthesis on heart rate variability spanning the continuum of time. Neurosci. Biobehav. Rev..

[6] Laborde S., Mosley E., Mertgen A. (2018). Vagal Tank theory: The three Rs of cardiac vagal control functioning—Resting, reactivity, and recovery. Front. Neurosci..

[7] Schneider C., Hanakam F., Wiewelhove T., Döweling A., Kellmann M., Meyer T., Pfeiffer M., Ferrauti A. (2018). Heart rate monitoring in team sports-A conceptual framework for contextualizing heart rate measures for training and recovery prescription. Front. Physiol..

[8] Abhishekh H.A., Nisarga P., Kisan R., Meghana A., Chandran S., Raju T., Sathyaprabha T.N. (2013). Influence of age and gender on autonomic regulation of heart. J. Clin. Monit. Comput..

[9] Dobbs W.C., Fedewa M.V., MacDonald H.V., Holmes C.J., Cicone Z.S., Plews D.J., Esco M.R. (2019). The Accuracy of Acquiring Heart Rate Variability from Portable Devices: A Systematic Review and Meta-Analysis. Sport. Med..

[10] Giles D., Draper N., Neil W. (2016). Validity of the Polar V800 heart rate monitor to measure RR intervals at rest. Eur. J. Appl. Physiol..

[11] Caminal P., Sola F., Gomis P., Guasch E., Perera A., Soriano N., Mont L. (2018). Validity of the Polar V800 monitor for measuring heart rate variability in mountain running route conditions. Eur. J. Appl. Physiol..

[12] Gilgen-Ammann R., Schweizer T., Wyss T. (2019). RR interval signal quality of a heart rate monitor and an ECG Holter at rest and during exercise. Eur. J. Appl. Physiol..

[13] Brupbacher G., Straus D., Porschke H., Zander-Schellenberg T., Gerber M., Von Känel R., Schmidt-Trucksäss A. (2019). The acute effects of aerobic exercise on sleep in patients with depression: Study protocol for a randomized controlled trial. Trials.

[14] Cataldo A., Bianco A., Paoli A., Cerasola D., Alagna S., Messina G., Zangla D., Traina M. (2018). Resting sympatho-vagal balance is related to 10 km running performance in master endurance athletes. Eur. J. Transl. Myol..

[15] Peguero J.G., Lo Presti S., Perez J., Issa O., Brenes J.C., Tolentino A. (2017). Electrocardiographic Criteria for the Diagnosis of Left Ventricular Hypertrophy. J. Am. Coll. Cardiol..

[16] Drezner J.A., Fischbach P., Froelicher V., Marek J., Pelliccia A., Prutkin J.M., Schmied C.M., Sharma S., Wilson M.G., Ackerman M.J. (2013). Normal electrocardiographic findings: Recognising physiological adaptations in athletes. Br. J. Sports Med..

[17] Hernando D., Garatachea N., Almeida R., Casajús J.A., Bailón R. (2016). Validation of heart rate monitor Polar RS800 for heart rate variability analysis during exercise. J. Strength Cond. Res..

[18] Wasserman K., Hansen J.E., Sue D.Y., Stringer W.W., Sietsema K.E., Sun X.G., Whipp B.J. (2011). Principles of Exercise Testing and Interpretation: Including Pathophysiology and Clinical Applications.

[19] Tanaka H., Monahan K.D., Seals D.R. (2001). Age-predicted maximal heart rate revisited. J. Am. Coll. Cardiol..

[20] Pietrobelli A., Rubiano F., St-Onge M.P., Heymsfield S.B. (2004). New bioimpedance analysis system: Improved phenotyping with whole-body analysis. Eur. J. Clin. Nutr..

[21] Noonan V., Dean E. (2000). Submaximal Exercise Testing: Clinical Application and Interpretation. Phys. Ther..

[22] Shaffer F., Ginsberg J.P. (2017). An Overview of Heart Rate Variability Metrics and Norms. Front. Public Health.

[23] Gore C.J., Booth M.L., Bauman A., Owen N. (1999). Utility of pwc75% as an estimate of aerobic power in epidemiological and population-based studies. Med. Sci. Sports Exerc..

[24] Batcho C.S., Thonnard J.L., Nielens H. (2012). PWC 75%/kg, a fitness index not linked to resting heart rate: Testing procedure and reference values. Arch. Phys. Med. Rehabil..

[25] Hillreiner A., Baumeister S.E., Sedlmeier A.M., Finger J.D., Schlitt H.J., Leitzmann M.F. (2019). Association between cardiorespiratory fitness and colorectal cancer in the UK Biobank. Eur. J. Epidemiol..

[26] Finger J.D., Gößwald A., Härtel S., Müters S., Krug S., Hölling H., Kuhnert R., Bös K. (2013). Measurement of cardiorespiratory fitness in the German Health Interview and Examination Survey for Adults (DEGS1). Bundesgesundheitsblatt Gesundheitsforsch. Gesundheitsschutz.

[27] Martínez J.P., Almeida R., Olmos S., Rocha A.P., Laguna P. (2004). A Wavelet-Based ECG Delineator Evaluation on Standard Databases. IEEE Trans. Biomed. Eng..

[28] Hernando D., Bailón R., Almeida R., Hernández A. QRS detection optimization in stress test recordings using evolutionary algorithms. Proceedings of the XLI Int Conf Computing in Cardiology.

[29] Mateo J., Laguna P. (2003). Analysis of heart rate variability in the presence of ectopic beats using the heart timing signal. IEEE Trans. Biomed. Eng..

[30] Bailón R., Laouini G., Grao C., Orini M., Laguna P., Meste O. (2011). The integral pulse frequency modulation model with time-varying threshold: Application to heart rate variability analysis during exercise stress testing. IEEE Trans. Biomed. Eng..

[31] Tomczak M., Tomczak E. (2014). The need to report effect size estimates revisited. An overview of some recommended measures of effect size. Trends Sport Sci..

[32] Cohen J. (1992). A power primer. Psychol. Bull..

[33] Lin L.I.-K. (1989). A Concordance Correlation Coefficient to Evaluate Reproducibility. Biometrics.

[34] Koo T.K., Li M.Y. (2016). A Guideline of Selecting and Reporting Intraclass Correlation Coefficients for Reliability Research. J. Chiropr. Med..

[35] Sanson A., Letcher P., Smart D., Prior M., Toumbourou J.W., Oberklaid F. (2009). Associations between early childhood temperament clusters and later psychosocial adjustment. Merrill. Palmer. Q..

[36] Gomez-Bruton A., Arenaza L., Medrano M., Mora-Gonzalez J., Cadenas-Sanchez C., Migueles J.H., Muñoz-Hernández V., Merchan-Ramirez E., Martinez-Avila W.D., Maldonado J. (2019). Associations of dietary energy density with body composition and cardiometabolic risk in children with overweight and obesity: Role of energy density calculations, under-reporting energy intake and physical activity. Br. J. Nutr..

[37] Buchheit M. (2014). Monitoring training status with HR measures: Do all roads lead to Rome?. Front. Physiol..

[38] Kleiger R.E., Stein P.K., Bigger J.T. (2005). Heart rate variability: Measurement and clinical utility. Ann. Noninvasive Electrocardiol..

[39] Michael S., Graham K.S., Oam G.M.D. (2017). Cardiac autonomic responses during exercise and post-exercise recovery using heart rate variability and systolic time intervals-a review. Front. Physiol..

[40] He W., Goodkind D., Kowal P. (2016). U.S. Census Bureau, International Population Reports. U.S. Gov. Publ. Off..

[41] Harridge S.D.R., Lazarus N.R. (2017). Physical activity, aging, and physiological function. Physiology.

[42] Ortega F., Cadenas-Sanchez C., Lee D., Ruiz J., Blair S., Sui X. (2018). Fitness and Fatness as Health Markers through the Lifespan: An Overview of Current Knowledge. Prog. Prev. Med..

[43] (2019). Polar Research Center Polar H10 Heart Rate Sensor System. White Pap..

